# Characteristics of top management team and Chinese tax planning nexus: Findings from a fuzzy-set qualitative comparative analysis

**DOI:** 10.3389/fpsyg.2022.964278

**Published:** 2022-07-25

**Authors:** Haiming Jiang, Eunyoung Kim

**Affiliations:** ^1^School of Knowledge Science, Japan Advanced Institute of Science and Technology, Nomi, Japan; ^2^School of Management, Dalian Polytechnic University, Dalian, China

**Keywords:** top management team characteristics, tax planning, the fuzzy-set qualitative comparative analysis, configuration, values and cognition

## Abstract

A top management team (TMT) has been identified as one of the key factors driving changes in tax planning strategy. Based on upper echelons theory, this study investigates whether configurations of TMT characteristics influence enterprise tax planning strategy by using the fuzzy-set qualitative comparative analysis (fsQCA). Using a panel data of China public companies, we found three configurations conducive to aggressive tax planning and two combinations conducive to low tax planning. Specifically, the level of education, tenure, age, career experience, and size of the top management team affect a firm’s propensity to engage in tax planning. Further, the results show that top management teams are not alike and highlight the differences in how these characteristics combine to impact tax planning.

## Introduction

Tax planning is often regarded as crucial for corporation operation. Corporates engage in various tax planning strategies to reduce the tax burden, pursuing a direct increase in after-tax returns and cash flow. However, any corporate tax planning activities can reduce the government’s tax collection. On the other hand, for firms, tax planning is costly, and inappropriate tax planning is adverse for firms. Because firms may either face with penalties from tax authorities or lose of reputation. Most recently, tax avoidance has been proved to be controversial with the CSR (Emerson et al., 2020; Mohanadas et al., 2020; Ding et al., 2022). The aggressive tax avoidance has cut down the public revenue significantly (OECD, 2016). Therefore, firms should consider the value generated not only for the shareholders but also for other stakeholders in their management strategies. In view of this, identification the determinants of aggressive tax planning have been paid attention by scholars. For instance, the firm-level characteristics are found associated with the extent of tax planning, including the scale of international operations, the compensation structure, and ownership patterns (Rego, 2003; Slemrod, 2004; Desai and Dharmapala, 2006). Furtherly, however, the variation in tax planning have yet to be explained completely. Scholars explore the determinants of firms’ tax strategies by introducing the upper echelons theory.

The upper echelons theory has been used in research relate to economics, management, and accounting, etc. According to the upper echelons theory, the observable demographic characteristics of management background are predicted to affect an organization’s strategies and performance (Hambrick and Mason, 1984), and abundant evidence has been obtained in several research areas. For example, TMTs’ career experience, age, tenure, gender composition, compensation, and education differentially shape managers’ strategic choices. These observable characteristics reflect the decision makers’ preference for risk-taking and capabilities in handling complex affairs within a firm. Based on this theory, attention toward the impact of TMTs’ characteristics on firm strategies increases and abundant evidence are found. Accordingly, as vital strategies in field of financial management, tax planning strategies might also be influenced by the backgrounds of TMT members. For example, the CEO, CFO, and other top executives affect tax aggressiveness individually (Dyreng et al., 2010; Francis et al., 2015). However, research on the association between a TMT and tax planning is rarely. Wahab (2020) conducts a multiple regression analysis to figure out how the TMT characteristics explain a firm’s tax planning strategies. The author includes age, tenure, education level, and gender in the regression model based on the existing theories. The results report that tenure and education of TMTs explain tax planning and permanent differences negatively, while tenure affects statutory tax rates positively. Although the author also predicts the potential impacts on tax planning of other characteristics such as age and gender, limit evidence is found. The traditional methods (e.g., multiple regression model) offer a single dominant “net-effect” explanation on the impact of TMT characteristics on tax planning. However, the effect may exist in a minority of cases that fit the relationship, and in other cases, the independent variable does not affect the dependent variable. Moreover, the configurations or recipes of constructs are better understood from the perspective of set-theoretic relations rather than correlation (Fiss, 2007). Thus, the present study builds on the upper echelons theory, and examines how five characteristics of top managers (education level, tenure, age, career experience, and size) explain firms’ aggressive tax planning or low level of tax planning. This study explores a panel of China firms over 2013 to 2019 after the corporate tax reform and adopts a fuzzy-set qualitative comparative analysis (fsQCA) to examine the determinants of aggressive tax planning (High_TP) and low-level tax planning (Low_TP).

## Literature review

As discussed in previous section, the roots of the relations between the backgrounds of TMTs with tax planning strategies go back to the upper echelons theory. Hambrick and Mason (1984) propose upper echelons theory as follows: “organizational outcomes, both strategies, and effectiveness, are viewed as reflections of the values and cognitive bases of powerful actors in the organization” (p. 193). The authors also argue that strategic choices and performance levels are partially predicted by managerial background characteristics such as age, education, socioeconomic background, financial position, gender, and group heterogeneity. Henceforth, the literature has focused more on the impacts of top management characteristics on firm policy choices with convincing evidence (Bertrand and Schoar, 2003; Bamber et al., 2010; Graham et al., 2013; Jiang et al., 2022; Zhou et al., 2022). Research in top management has identified TMTs as the primary driver of a firm’s strategic decision-making (Papadakis and Barwise, 2002; Hambrick, 2007). For example, Bantel and Jackson (1989) document that TMT characteristics such as education level and age diversity impact product performance. TMT composition has been found to affect a firm’s performance in the stock market (Pollock et al., 2010). Likewise, investigating the characteristics of TMT as a driving factor in firms’ tax planning choices is important in extending the upper echelon theory and enriching research on the determinants of tax planning choices. Therefore, investing the characteristics of TMT that can explain tax planning strategies is valuable to shed light on preferences of tax planning strategies from the internal governance perspective. The next subsections explore how these factors determine tax planning.

While TMT members are unlike to be directly engaged in the tax aggressiveness, their traits and experiences allow them to use their authority to lead tax planning process by communicate with and integrate information from various firm function (Plečnik and Wang, 2021). Tax authorities entitle corporates to arrange tax plans in many countries. When firms face tax planning choices, the background of the TMT may lead to different levels of tax planning. Specifically, management traits, such as the education level, tenure, age, expertise background, and size are predicted to impact top management’s tax oversight upon the extant theories and empirical evidence.

This study contributes to the literature in three ways. First, the extant studies focus on the impact of TMT background individually (Dyreng et al., 2010). Although Plečnik and Wang (2021) document TMT intrapersonal functional diversity affect tax avoidance (or tax aggressiveness), few literature focus on the impact of TMT traits on tax planning in comparison to individuals. Second, this study contributes to the strand of accounting literature identifying combinations of characteristics as determinants of tax planning. This finding is different from prior literature in accounting which is concerned with the single characteristic and rarely discuss the value of synthetic characteristics. Third, the traditional symmetric (e.g., regression or the structural equation modeling) methods test relationships between each characteristic and dependent variables to explain the linear additive impact on focal outcomes. Specifically, each independent variable is considered discretely, that is, holding constant the impact of all other independent variables on the dependent variable, except for a limited number of interactive terms that may be included in the analysis to reflect the hypothesized interdependence of two or more antecedent variables. While this study focuses on the causal recipes, by examining with-case relationships among top management team characteristics and characterize cases as having a particular combination of characteristics that associates with the tax planning strategies. In this regard, this study fills this gap theoretically and quantitatively.

## Research design

**FIGURE 1:**
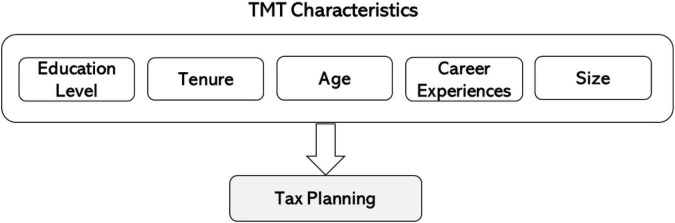
The conceptual framework.

Previous studies have identified that education level, tenure, age, career experience, and size of the TMT are related to corporate strategies. Figure 1 shows a conceptual framework for the ways in which traits may drive tax planning, where tax planning results from the complex interactions between these characteristics. In the conceptual framework in Figure 1, each condition has the potential to drive the tax planning either by itself, or in combination with other conditions (or just single condition). There is likely to be more than one causal combination explaining a firm’s engagement in tax planning. The following subsections highlights the potential effect on tax planning of these traits.

### Top management team education level and tax planning

Education indicates knowledge and skills (Hambrick and Mason, 1984). Like work experience, educational background has been identified as one of the key factors influencing how TMTs make firm decisions (Hambrick and Mason, 1984; Hitt and Tyler, 1991; Wiersema and Bantel, 1992). Top management’s education level is positively associated with innovation (Becker, 1970; Kimberly and Evanisko, 1981). Kimberly and Evanisko (1981) posit that TMTs with members who have high formal education backgrounds are positively engaged in innovation in both management and technologies. Likewise, Bantel and Jackson (1989) conduct an empirical test on a sample derived from the banking industry and find that management with higher education levels embraces innovation to a greater extent. Lee et al. (2017) also suggest that TMT members’ educational background affects a firm’s proportion of exploratory research and development (R&D) activities. Additionally, TMT members’ education is positively related to changes in strategies (Wiersema and Bantel, 1992). In conclusion, high formal education may be a signal of management’s ability to process information, and this ability makes it easier to deal with complex situations within a firm. Regarding the association with tax planning, this would be more complicated. On one hand, highly educated members may expect to obtain the benefit of tax planning, which leads to more aggressive tax avoidance. On the other hand, tax avoidance brings incremental costs such as potential lawsuits and reputational risks. Thus, TMTs with a high educational level diversity may hesitate to engage in more tax planning.

### Top management team tenure and tax planning

Top management team tenure is one of the most attractive attributes of TMT. Firms’ decisions are not only impacted by top management’s work experience and education. TMT members’ tenure in the organization can also affect their decisions regarding operations and strategy choices (Hambrick and Mason, 1984; Bantel and Jackson, 1989; Chen et al., 2010). The longer the TMT members’ average tenure, the similar their perceptions and decisions the TMT members hold. Additionally, TMT tenure can improve internal communication efficiency. Similarly, Katz and Allen (1988) posit that the comparatively long tenure of the TMT would generate stability of the team, as well as the degree of socialization.

Existing research links TMT tenure to firm performance, coordination, and social cohesion. For example, Sun et al. (2006) find a positive relationship between the average tenure of the TMT members and the firm performance. Eisenhardt (1989) concludes that long-term cooperation enables members to better understand how to communicate and cooperate with other members among a team. Likewise, Michel and Hambrick (1992) suggest that the long tenure of TMT members promotes integrity and opportunities for managerial value judgment.

It has been proven that a firm’s R&D-related decisions are influenced by TMT tenure in the organization. In particular, when the TMT consists of members with relatively short tenures, it is less probable for the TMT to support large resource-consuming R&D projects, such as explorative R&D (Hambrick, 2007).

However, top management with short tenure may feel stressed in exhibiting their values and abilities in the short term in an organization (Chen et al., 2010). Thus, it is expected that a TMT with a shorter average tenure will exhibit more aggressive tax planning to demonstrate its abilities. Based on these analyses, the association between TMTs’ tenure and tax avoidance can be equivocal. A TMT with a longer tenure might be involve in more tax planning, as they have more firm-specific knowledge and experience to gauge potential benefits of tax planning practices.

### Top management team age and tax planning

Hambrick and Mason (1984) posit that firms with young managers are more inclined to pursue risky strategies than those with older managers. Risky strategies include unrelated diversification, product innovation, and financial leverage. Lai and Liu (2018) posit that the average age of TMT members, education, and career experience impact firms’ over-investment strategies. Kumar (2019) investigates the relationship between the age demographic of a TMT and a firm’s environmental management strategy, comprising compliance only and beyond-compliance initiatives. As expected, the author finds that aging TMTs support beyond-compliance in comparison to compliance-only environmental management strategies. Tanikawa et al. (2017) reveal that the age diversity of the TMT attenuates firm performance.

Wiersema and Bantel (1992) argue that flexibility and risk-bearing capacity decrease with age. Similarly, Wayde et al. (2017) posit that older executives tend to make decisions following a familiar pattern. For example, an older executive may be more conservative in corporate decision making. Plausible explanations include: first, older management’s ability to integrate information in making decisions may be weaker than younger executives (Taylor, 1975); second, older executives may be near retirement, so they have shaped expectations about future lives; thus, they will intentionally avoid risky choices (Carlsson and Karlsson, 1970).

Positively, older managers are typically risk-reverse, one may expect they engage in less tax planning. However, a TMT with older members may generate more aggressive tax planning due to the members’ stronger cognitive ability to predict benefits that can offset potential costs (Wahab, 2020). Thus, the effect of TMT age on tax avoidance may be mixed.

### Top management team career experiences and tax planning

The observable demographic characteristics of management backgrounds have been favored in several areas of research. For example, career experiences differentially shape managers’ strategic choices. Empirical research confirms that managers seek strategies that are in line with their work experience (Smith and White, 1987). Top managers with technical financial functions are conventional, orderly, and overcautious, suggesting that they may adopt conservative tax planning. Similarly, finance managers pursue administrative complexities. Managers with legal backgrounds are more sensitive to litigation risk. Therefore, managers’ accounting, financial, and legal career experiences affect their preferences (Hambrick and Mason, 1984; Jensen and Zajac, 2004). Bamber et al. (2010) document that managers promoted from legal backgrounds hold greater sensitivity to litigation risk; managers with accounting and finance are inclined to more precise disclosure types, indicating a conservative upcoming earnings prediction.

However, there is also research that explores how functional experiences exacerbate management’s risk strategy. For instance, Finkelstein (1992) finds that if a firm possesses a high proportion of powerful TMT members with financial backgrounds, it tends to adopt an acquisition strategy. Lee et al. (2017) state that TMT members with experience in R&D-related positions tend to focus on explorative R&D activities. In addition, top managers who possess experience working in R&D-related functions enhance their technological competitiveness (Daellenbach and McCarthy, 1999). In the tax planning context, managers who have functional backgrounds in accounting, finance, and law are more familiar with transaction-related regulations, and they may engage in tax avoidance to the maximum extent permitted by legislation. Thus, functional experiences have both beneficial and detrimental implications.

### Top management team size and tax planning

Top management team size affects the output of organizations (Smith and White, 1987). Haleblian and Finkelstein (1993) argue that there are several advantages to large TMTs. Specifically, they reveal that large top management teams perform better and that this finding is significant in an environment where top managers are allowed high discretion in making strategies. Michel and Hambrick (1992) find that firms facing bankruptcy have smaller management teams than matched-paired firms. Two studies have also investigated the positive association between firm growth and team size (Cooper and Bruno, 1977). The extant literature argues that large groups are more advantageous than small ones because the capabilities and resources of large teams are the strength to solve complicated tasks (Shull et al., 1972). Such capabilities and resources are helpful for high-quality decision making. These findings support Michel and Hambrick’s argument that the number of people results in a team’s resources being available to address problems.

However, large-sized groups also tend to face increasing coordination and communication problems. Small-sized groups have more cohesiveness, and their members’ satisfaction is greater. In addition, small teams spend less time reaching a consensus (Shull et al., 1972). Therefore, small groups may be suitable for organizations in which problem-solving tasks are relatively easy.

A large team performs well in complex decisions because it controls more resources. Concerning tax choices, such a TMT may compromise well in terms of risks and benefits. However, large firms have been found to use less tax planning, even given their large potential benefits and relatively small costs (Weisbach, 2002). This is because tax-aggressive firms may face significant reputational risk. To some extent, the cost of reputational risk may exceed the explicit costs. Therefore, the relationship between size and tax planning might be uncertain.

In summary, upper echelons theorize that a series of TMT characteristics affect organizations’ strategies and performances. Based on the above reasoning, the present study proposes the following proposition:

Proposition 1. Different combinations of TMT educational level, tenure, age, functional experience, and size are sufficient to predict high-level or low-level tax planning practices, but each condition alone is not.

## Data collection and measures

### Data collection

The sample in this study comprises Chinese A-share main public firms listed on the Shanghai Stock Exchange and Shenzhen Stock Exchange, which were active for 7 years, beginning in 2013. The reasons for choosing this length of time include the following: first, it allows the calculation of long-term tax planning to identify firms engaging in tax policies that cut tax liabilities over the long term. Second, Chinese public firms were required to adopt the latest Generally Accepted Accounting Standards (GAAP), beginning January 01, 2008. In addition, the latest Corporate Tax Law of the People’s Republic of China took effect on January 01, 2008. However, firms that apply preferential tax rates have a 5-year interim to adopt the new standard income corporate tax rate (e.g., 25%). By 2013, all listed companies had applied standard corporate tax rates. Consequently, the accounting data used to measure the variables in the model are established consistently. The sample is constrained to firms for which tax aggressiveness measures can be calculated.

In the context of this study, TMT includes a firm’s CEO, CFO, COO, CTO, and heads of business units (Hambrick and Finkelstein, 1995). The data were collected from the China Stock Market and Accounting Research Database (CSMAR) and the study period runs from 2013 to 2019.

The sample was collected as follows: first, listed companies under special treatment were excluded. Second, companies in the financial industry were excluded to control for bias in reporting regulations. Third, observations were constrained to the measurement of TMT characteristics and tax avoidance. Fourthly, we omitted observations with a negative pre-tax income to avoid errors in the measure of tax planning. Finally, an unbalanced dataset containing 7,212 firm-year observations of 77 industries over a 7-year timespan (2013–2019) was obtained. All the variables are defined in Table 1.

**TABLE 1:** Variable measurements.

Variable	Description	Measurement
BTDs	Level of tax planning	BTDs = [pre-tax profit-(Income tax expense-deferred tax expense)/Nominal corporate tax rate]/beginning assets
TMT_edu	Education background of TMT members	The TMT members’ education degree is coded as follows: if a TMT member has a degree with special school or lower, 1 is coded; members with a degree of the institution are coded 2; members have a bachelor’s degree are coded 3; members have a master’s degree is coded 4; members have a Ph.D. degree is coded 5. Furthermore, we used the Herfindahl–Hirschman index (Hirschman, 1945; Herfindahl, 1950) to capture TMT education background. The index was calculated as 1−∑*Si*^2^, where *Si* is the percentage of TMT members in each of the educational background categories.
TMT_ten	Tenure of TMT members	The average tenure of TMT members in months
TMT_age	Age of TMT members	The average age of TMT members in years
TMT_exp	Expertise experience of TMT members	The proportion of members with financial, accounting, or legal experiences in a TMT. Each member of the TMT is coded with 1 if they have experience of working in accounting, financial, or legal, and 0 otherwise. The variable is measured by the proportion of TMT members coded 1 for each firm and observation year.
TMT_size	Size of the TMT	Number of TMT members

### Measures

#### Outcome: tax planning

Tax authorities in many countries recognize that taxpayers are entitled to arrange their affairs to reduce tax liability. However, Blouin (2014) argues that only those tax planning arrangements beyond acceptable, legislated, or observed tax deductions should constitute aggressive tax strategies. Therefore, the aggressiveness of a specific firm’s tax planning is best measured in comparison to the located industry. The GAAP and cash effective tax rates (ETRs) are often used in extant literature to capture firms’ tax aggressiveness (e.g., Dyreng et al., 2008). Prior studies also use measures based on firms’ likelihood of entering a tax shelter (Wilson, 2009; Lisowsky, 2010), tax haven activity (Dyreng and Lindsey, 2009), and discretionary permanent book-tax differences (Frank et al., 2009; Xu, 2021). However, these measures might be limited because, for some measures, specific conditions are needed for firms. However, there is no benchmark for a “normal” level. Although Balakrishnan et al. (2019) take the innovative method a step further, this method is not suitable for the sample in this study. As corporates are entitled to many tax reductions according to tax policies in China, errors may exist when measuring tax aggressiveness by ETRs (Xu, 2021); therefore, in this study, book-tax differences (BTDs) is used as a basic proxy of the firm’s level of tax planning. The calculation of the BTDs is presented in Table 1. A larger number of BTDs means aggressive tax planning, whereas a smaller number indicates less tax planning.

#### Conditions

As discussed in Section “Literature review,” the age, tenure, education level, expert experience, and size of a TMT are conditions of tax planning in this study. The measures of TMT characteristics mentioned in are listed in Table 1. All TMT characteristics data were obtained from the CSMAR database. In next sections, this study looks at the influence of TMT synthetic characteristics on tax planning of aggressive and non-aggressive.

## Fuzzy-set qualitative comparative analysis

The FsQCA method is based on complexity theory and use an inductive research method based on the principles of conjunction, equifinality, and causal asymmetry (Misangyi and Acharya, 2014). FsQCA builds on the idea that configurations or recipes of constructs are better understood in perspective of set-theoretic relations rather than correlation (Fiss, 2007). The objective of qualitative comparative analysis is to identify the different configurations of conditions linked to focal outcome (Ragin and Strand, 2008). The fsQCA provides a systematic analysis of data, revealing sufficient configurations of conditions to reach focal outcome. In brief, fsQCA has the perspective that cases are composed of combinations of theoretical related attributes (Misangyi and Acharya, 2014), that the relations between these attributes and the focal outcome can be explained by subsets (Ragin and Strand, 2008).

Although FsQCA initially is developed for small-sample research (Ragin, 2000), now it is applied across a range of recent studies have shown its potential for large sample organization studies (Guedes et al., 2016; Beynon et al., 2020; Mustafa et al., 2022; Yang et al., 2022). It is an especially effective method to social science research, because unlike traditional statistical analyses, fsQCA does not identify the independent effect of a variable on the likelihood of a focal outcome (Fiss, 2011). Thus, fsQCA has been widely used by public policy researchers for comparisons of outcomes in countries (Rihoux, 2013; Beynon et al., 2020). Usually, fsQCA studies use cross-sectional data and without incorporating temporal effects, but there also exist literature incorporate the time effect and conduct panel data analysis using fsQCA. Referring to García-Castro and Arino (2013), this study reveals the combinations of TMT characteristics that contribute toward high and low tax planning by using panel data.

### Calibration of set membership

As part of the preparation for fsQCA, the calibration of the conditions and outcome is required. This calibration transforms the original data (all continuous variable here) to fuzzy membership scores ranging from 0–1 to construct a continuous fuzzy set for each attribute (Ragin, 2008). The calibration applied here follows the direct method given by Ragin (2008), and a more detailed description outline by Andrews et al. (2016).

Firstly, following Greckhamer et al. (2013), the “lowest,” “highest,” and “surrounding 50th percentile” pairs of cases were identified and considered against the anchors in terms of the threshold for fully-out membership, the threshold for fully-in membership, and the crossover point, respectively. Then these anchors were used to construct the continuous fuzzy membership score ranging from 0 to 1. The established threshold values were then checked by the authors (see Andrews et al., 2016). Table 2 summarizes the fuzzy sets, including the calibration anchors and descriptive statistics for each fuzzy set.

**TABLE 2:** Calibration values and statistics.

	Statistics					Calibration values at			Fuzzy values descriptive			
			
	*N*	Mean	Std. dev	Min	Max	95%	50%	5%	Mean	Std. dev	Min	Max
**Outcome**												
BTDs	7212	0.016	0.029	−0.238	0.113	0.062	0.0154	−0.0184	0.497	0.305	0.01	0.99
**Conditions**												
TMT_edu	7212	0.278	0.239	0.056	1	1	0.2	0.095	0.582	0.268	0.05	0.98
TMT_ten	7212	48.119	17.407	10.194	99.696	80.316	46.333	22.556	0.513	0.303	0.01	0.99
TMT_age	7212	47.789	3.375	38.5	55.6	53.200	47.894	42.067	0.497	0.305	0.01	0.99
TMT_exp	7212	0.372	0.184	0	1	0.75	0.333	0.125	0.510	0.298	0.01	0.99
TMT_size	7212	6.968	2.577	3	18	12	7	4	0.572	0.302	0	0.98

### Necessity analyses for tax planning

The analysis of necessary conditions in fsQCA is an independent procedure to examines whether individual conditions (or one condition) may be necessary for the outcome to occur (Ragin, 2008). For necessity to hold for a firm-year observation, the membership score on the outcome must be consistently lower than the membership score of the condition under consideration. A condition (or combination of conditions) is necessary if “it is present in all instances of an outcome” (Ragin, 2000, p. 203); a condition is necessary if the outcome occurs whenever that condition occurs, though the outcome may occur at presence of other conditions (Ragin, 2008).

Given the asymmetry of fsQCA, results for the two outcomes (e.g., High_TP and Low_TP) are presented in Tables 3, 4. According to Ragin (2008), a condition could be deemed as necessity only if it exceeds the 0.90 consistency threshold and has non-trivial coverage. The necessity analyses on all the conditions demonstrated that no condition meets the criteria, so no necessary conditions exist.

**TABLE 3:** Overview of necessary conditions for High_TP.

Conditions	Cons	Cov
TMT_edu	0.732	0.426
∼TMT_edu	0.526	0.513
TMT_ten	0.685	0.691
∼TMT_ten	0.729	0.490
TMT_age	0.611	0.459
∼TMT_age	0.719	0.441
TMT_exp	0.629	0.621
∼TMT_exp	0.652	0.630
TMT_size	0.656	0.585
∼TMT_size	0.529	0.669

∼ represents the absence of a condition.

**TABLE 4:** Overview of necessary conditions for Low_TP.

Conditions	Cons	Cov
TMT_edu	0.691	0.496
∼TMT_edu	0.582	0.568
TMT_ten	0.751	0.609
∼TMT_ten	0.784	0.638
TMT_age	0.588	0.419
∼TMT_age	0.652	0.645
TMT_exp	0.659	0.598
∼TMT_exp	0.686	0.655
TMT_size	0.662	0.465
∼TMT_size	0.645	0.539

∼ represents the absence of a condition.

### Sufficiency analyses

The role of configurations of conditions in understanding High_TP and Low_TP is considered conducting sufficiency analysis. Sufficiency analysis seeks to find distinct recipes of attributes that meet certain criteria of sufficiency for the outcome to occur. For sufficiency to hold for a firm-year observation, the membership score of the outcome must be consistently higher than the membership score of the combination of conditions.

Sufficiency analyses begin with the use of a truth table algorithm, aiming to map the logically possible and empirically occurring combinations of fuzzy sets, and the outcome (either High_TP or Low_TP). While five conditions considered here, there are 2^5^ = 32 logically possible configurations to consider. The configurations are characterized by 0 and 1 values across the five conditions. Where 0 indicates the absence and 1 denotes the presence of each condition. Each reported configuration is described by several relevant values, including the number of firms belonging to each configuration in strong membership, the level of consistency measured as the degree to which it can be shown that membership in the outcome is consistently less than equal or equal to membership in the cause (Ragin, 2008). Then further consideration is given to those assured configurations which have association with either High_TP or Low_TP. Two more thresholds must be set: frequency and consistency. Frequency is the minimum number of firm-year observations for each configuration; consistency is the minimum consistency level for each recipe (Ragin, 2008). The priori minimum thresholds for consistency and the frequency of cases per configuration is 0.85 and 10. The threshold values were the same for High_TP and Low_TP. Next, sufficiency analyses were conducted to identify combinations of conditions. These combinations, termed causal recipes, will lead to the occur of focal outcomes.

Tables 5, 6 depict the results of sufficiency analysis. Three and two causal recipes were identified separately to explain each outcome (e.g., High_TP or Low_TP). The findings in Table 5 show that the solution is informative with a consistency value of 0.850 and coverage of 0.395. Table 6 reports a consistency value of 0.814 and coverage of 0.478. These values are higher than the minimum acceptable thresholds (e.g., 0.8 for consistency), following the recommendation of Ragin (2008) and Woodside (2013).

**TABLE 5:** Results of both the parsimonious and intermediate solution of High_TP.

Casual configuration		Raw coverage	Unique coverage	Consistency
1	∼TMT_age*∼TMT_edu*∼TMT_size	0.301	0.053	0.874
2	∼TMT_age*∼TMT_size*∼TMT_ten*∼TMT_exp	0.284	0.064	0.879
3	TMT_ten*∼TMT_exp*∼TMT_size*∼TMT_edu	0.249	0.029	0.902

Solution coverage: 0.395.

Solution consistency: 0.850.

∼ represents the absence of a condition.

**TABLE 6:** Results of both the parsimonious and intermediate solution of Low_TP.

Casual configuration		Raw coverage	Unique coverage	Consistency
1	TMT_age*TMT_exp *TMT_edu	0.450	0.136	0.824
2	TMT_age*TMT_exp*TMT_ten*TMT_size	0.342	0.028	0.865

Solution coverage: 0.478.

Solution consistency: 0.814.

∼ represents the absence of a condition.

Considering High_TP first, there were three pathways conducive to High_TP, as shown in Table 5. The configuration (HTP1) with the highest coverage (0.301) and good consistency (0.874) is the absence of TMT_age, TMT_edu, or TMT_size. The second configuration (HTP2) indicates that the absence of TMT_age, TMT_ten, TMT_exp, or TMT_size is conducive to engagement in tax aggressiveness. The third configuration (HTP3) shows the absence of TMT_exp, TMT_size, or TMT_edu leads to more tax avoidance when combined with the presence of TMT_ten. The core condition, TMT_size, exists in all recipes in the form of absence.

Considering the low level of tax planning, two configurations were found conducive to Low_TP, as shown in Table 6. The configuration (LTP1) with higher coverage (0.450) and good consistency (0.824) is the presence of TMT_age, TMT_exp, and TMT_edu. Another configuration (LTP2) shows that the presence of TMT_age, TMT_size, TMT_exp, and TMT_size is sufficient for less tax planning.

In sum, TMT_age, TMT_size, TMT_edu, and TMT_exp are included in High_TP configurations in the form of absence, while they are included in Low_TP configurations in the form of presence, indicating that they affect tax planning symmetrically. While TMT_ten does not affect tax avoidance symmetrically since it exists in High_TP configurations in different manners (both absence and presence).

## Discussion

The present study explores how different combinations of top management’s characteristics explain firms’ tax planning. We found three configurations conducive to aggressive tax planning and two combinations conducive to low tax planning.

For High_TP, three configurations were found. One recipe indicates that firms with a younger, smaller TMT have a propensity to engage in more aggressive tax planning when they have a similar education level. Likewise, a small TMT, comprised of younger members with shorter tenure and fewer expert experiences, creates higher risk by engaging in more aggressive tax planning. Moreover, a small TMT that has fewer expert members and a similar education level is inclined to be more tax aggressive when its members have a longer tenure.

Looking at Low_TP, two configurations were found. One recipe shows that an older TMT, with a higher proportion of expert experience and diverse education levels, focuses less on tax planning. The other recipe indicates that an older TMT, with longer tenure and higher level of expert experience also pays less attention on tax planning when the TMT is big. The two combinations of characteristics have two same core conditions: age and expert experience. Substitute conditions are the education level, tenure and size. In both configurations, age and expert experience exist in the form of presence. While these two conditions emerge in positive configurations in the form of absence.

Consistent with the Proposition 1, the casual conditions alone are neither sufficient nor necessary for tax planning practices. They combine with each other to achieve various levels of tax avoidance.

Overall, the results show that the consistency is significant for the panel taken as a whole, the coverages, in general, indicate that the explanatory power of configurations of TMT characteristics about tax planning is acceptable.

## Robustness analysis

This study conducts several robustness tests to check the validity of the results. First, the study uses alternative calibration values: 0.90, 0.50, and 0.10. The results of both the parsimonious and the intermediate solution exhibit the same configurations with a slight increase in consistency and coverage. On the other hand, we also measure the tax planning using the cash effective tax rate, the conclusions keep unchanged.

## Conclusion, limitations, and implications

### Conclusion

A great majority of upper echelons theory has been conceptualized to be tested using symmetric quantitative methods, such as multiple regression analysis and structural equation modeling. These traditional symmetric methods test relationships between explanatory variables and independent variables to explain the strategies of top management teams. However, symmetric methods require the data to conform to restrictive assumptions, including distributed data, symmetric data relationships, and independence of the variables, and these restrictions limit the ability of these approaches to explain the complex management strategies. Using single trait of TMTs to explain firm strategies might be incomplete, and synthetic characteristics would better explain a firm’s tax planning strategy.

As an echo to Woodside’s (2013) calling that moving beyond relying on the dominant logic of multiple regression analysis, this study uses the fsQCA in testing upper echelons theory. This study presents a novel method to understand the strategy choices of top management teams. It answers which configurations of situational determinants impact tax planning. Specifically, the findings indicate that the characteristics of TMTs matter in tax planning choices. Education level, tenure, age, expert experience, and size of a TMT affect a propensity of engaging in tax planning.

Our study provides some evidence that differs from the extant literature. For instance, according to Wahab’s (2020) findings, tenure and education level are negatively related to tax planning. Our findings partially support the results, since education level is included in recipes conducive to high-level tax avoidance in the form of absence, while it is included in combinations conducive to low-level tax avoidance in the opposite manner. However, we found conflicting evidence regarding the influence of tenure on tax avoidance. As shown in Table 5, both the absence and presence of tenure could result in aggressive tax planning when combined with other conditions. Furthermore, the author links age to tax planning. Although the direction of age is negative, such a relationship is not significant. However, our results support the effect of TMT age on tax avoidance when combined with other conditions. Moreover, we found that the effect of age is symmetric, since it exists in two combinations of high-level tax planning in the form of absence, while it exists in both combinations of low-level tax planning in the form of presence.

Considering size, it presents in combinations conducive to aggressive tax planning in the form of absence but exists in the form of presence in configurations regarding Low_TP. This finding indicates that a large TMT is inclined to engage in less aggressive tax planning. Thus, our finding is consistent with Deslandes et al.’s (2020) finding that a larger audit committee plays an important role in constraining tax aggressiveness.

The study explores research in the fields of upper echelons theory and tax planning by finding different paths for each result (e.g., High_TP and Low_TP). Distinct bundles of TMT characteristics associated with tax-planning strategies are identified.

Theoretically, these findings provide further evidence of upper echelons theory and taxation area. Practically, the study provides useful insights for board committees to appoint top management team members pursuing healthy performance and moderate tax risk. The configurations conducive to Low_TP also indicate the way of taking more social responsibilities.

Finally, this study is valuable to investors, creditors, analysts, and auditors, as it serves as a reminder that the combinations of a TMT’s traits need to be considered when making decisions.

This study may serve as a starting point for introducing algorithms to investigate patterns and test theories in the fields of accounting and management. Moreover, there are many research opportunities in the field of accounting and taxation using fsQCA.

### Limitations

Surely, fsQCA has limitations. It is an inductive and iterative method that based on specified cases, to seek to combinations of theoretical conditions on focal outcome (Greckhamer et al., 2018). It could not explain how and why those combinations lead to focal outcomes. Secondly, the results are cases dependent because most steps in fsQCA rely on the researchers’ judgments. For example, the choices of representative sample and the conditions, the setting of three anchors for independent and dependent variables, and the chosen of the thresholds of the consistency and coverage. Thirdly, the results of fsQCA are sample-dependent, configurations derived from different sample may be distinct. Therefore, the reproducibility and validity of fsQCA results might be a question to be resolved. Fourthly, a warning has to do with the issue of causality. Although conceptually the arguments in the current study assume causality (e.g., that characteristics of a TMT lead to aggressive tax planning), the investigation on cross-sectional, and configurational approach generally do not allow for the claims of the causal relation. Therefore, future configurational research that addresses this issue is needed. Finally, as this study focuses only on large, listed firms, extending the findings to other categories of firms could be limited.

## Data availability statement

The original contributions presented in this study are included in the article/supplementary material, further inquiries can be directed to the corresponding author.

## Author contributions

HJ conceived and designed the study and drafted the manuscript. EK guided the research and made the final proofreading. Both authors contributed to the article and approved the submitted version.

## Conflict of interest

The authors declare that the research was conducted in the absence of any commercial or financial relationships that could be construed as a potential conflict of interest.

## Publisher’s note

All claims expressed in this article are solely those of the authors and do not necessarily represent those of their affiliated organizations, or those of the publisher, the editors and the reviewers. Any product that may be evaluated in this article, or claim that may be made by its manufacturer, is not guaranteed or endorsed by the publisher.
